# Early Root Transcriptomic Changes in Wheat Seedlings Colonized by *Trichoderma harzianum* Under Different Inorganic Nitrogen Supplies

**DOI:** 10.3389/fmicb.2019.02444

**Published:** 2019-10-25

**Authors:** M. Belén Rubio, A. Emilio Martínez de Alba, Carlos Nicolás, Enrique Monte, Rosa Hermosa

**Affiliations:** ^1^Department of Microbiology and Genetics, Spanish-Portuguese Institute for Agricultural Research (CIALE), University of Salamanca, Salamanca, Spain; ^2^Department of Botany and Plant Pathology, Spanish-Portuguese Institute for Agricultural Research (CIALE), University of Salamanca, Salamanca, Spain

**Keywords:** Wheat Genome Array, plant defense, plant growth, *Trichoderma*, chemical fertilization, nitrogen use

## Abstract

Wheat is one of the most important crops worldwide. The use of plant growth promoting microorganisms, such as those of the genus *Trichoderma*, constitutes an alternative to chemical fertilizers, since they are cheaper and are not detrimental to the environment. However, the interaction between *Trichoderma* and wheat plants has been scarcely studied, at least at a molecular level. In the present work, a microarray approach was used to study the early transcriptomic changes induced in wheat roots by *Trichoderma harzianum*, applied alone or in combination with different concentrations of calcium nitrate [Ca(NO_3_)_2_], which was last used as nitrogen (N) source. Our results show that *T. harzianum* causes larger transcriptomic changes than Ca(NO_3_)_2_ in wheat roots, and such changes are different when plants are challenged with *Trichoderma* alone or treated with a combination of *T. harzianum* and Ca(NO_3_)_2_. Overall, *T. harzianum* activates the expression of defense-related genes at early stages of the interaction with the roots, while this fungus reduces the expression of genes related to plant growth and development. Moreover, the current study in wheat roots, subjected to the different *T. harzianum* and Ca(NO_3_)_2_ combinations, reveals that the number of transcriptomic changes was higher when compared against those caused by the different Ca(NO_3_)_2_ concentrations than when it was compared against those caused by *T. harzianum*. N metabolism gene expression changes were in agreement with the levels of nitrate reductase activity measured in plants from *Trichoderma* plus Ca(NO_3_)_2_ conditions. Results were also concordant with plant phenotypes, which showed reduced growth at early interaction stages when inoculated with *T. harzianum* or with its combination with Ca(NO_3_)_2_ at the lowest dosage. These results were in a good agreement with the recognized role of *Trichoderma* as an inducer of plant defense.

## Introduction

Wheat (*Triticum aestivum* L.) is one of the most important crops on Earth, with an area harvested of 220 million ha and a global production of 770 million tons, China and India being the major producers followed by Russia and United States (FAOSTAT, 2018). It is considered to be the most important food grain source for humans, providing about a 19% of the total calories consumed by humans, as well as representing a 20% of proteins in the human diet (Botha et al., 2017; International Wheat Genome Sequencing Consortium [IWGSC], 2018). Wheat flour is used for the production of bread, pasta, biscuits and other bakery and industrial products.

Nitrogen (N) is the most important nutrient for plants and, after water, is the major limiting factor for their growth and development. Thus, in modern extensive agriculture, the application of N fertilizer is a common practice, aiming to increase production and achieve maximum yields in many crops (Rockström et al., 2009). There is an absolute requirement of N for wheat growth, and crop yield and quality depend upon substantial N inputs (Vicente et al., 2016; Zörb et al., 2018). However, it is well known that the most diffused cereal crops, like wheat, rice and maize, use only 30–40% of the applied N fertilizers, while the rest remains unused causing severe environmental pollution (Curci et al., 2017). It has been reported that the human processes -primarily the manufacture of fertilizer for food production- have converted around 120 million tons of N_2_ from the atmosphere per year into reactive forms, and most of them end up polluting the environment. Thus, this value has overcame by four times the capacity of recycling that our planet possesses (Rockström et al., 2009). The H2020 program of the EU has suggested a reduction of N fertilization to reach a limit of 170 kg/ha/year. However, currently in Spain, 2.4 million ha are devoted to wheat crops employing 200 kg/ha/year. This drastic situation is a suitable scenario for developing novel strategies to increase crop yields. This need has led to the search for biostimulants for plant nutrition as biological and environmentally friendly alternatives to reduce or substitute the use of inorganic fertilizers, while ensuring the yield and high quality of crops (Vita et al., 2018).

Despite the fact that wheat is economically one of the most important crops, until quite recently, there have not been many tools available for analyzing the molecular mechanisms of gene expression. Most probably due to the vast size of the wheat genome (about five times larger than the human genome or 40 times than the rice genome) and its high content of repetitive highly sequences, as a consequence of being a hybrid of three highly similar subgenomes (International Wheat Genome Sequencing Consortium [IWGSC], 2018). Early transcriptomic studies used 8K wheat microarrays derived from expressed sequence tag (EST) clones. These initial tools allowed to partially study the response of the wheat plant to pathogens, such as *Fusarium graminearum* (Golkari et al., 2007, 2009), or to abiotic stresses such as low temperature (Laudencia-Chingcuanco et al., 2011). The availability of 61K commercial microarrays enabled analysis of around a half of the wheat genome, which represented a seven-fold increase when compared with previous microarray versions (Brenchley et al., 2012). For instance, wheat plant responses to a herbicide have been analyzed using that tool (Pilcher et al., 2017). Nowadays, up to 94% of wheat genome has been covered and 107,891 gene models proposed (Brenchley et al., 2012; International Wheat Genome Sequencing Consortium [IWGSC], 2018), facilitating genome-wide studies. In fact, a recent study has employed RNA-seq technology to analyze the transcriptomic response of wheat plants to N starvation (Curci et al., 2017). RNA-seq is currently the most advantageous and economical tool for exploring genomes, as it does not suffer from hybridization-based limitations and has demonstrated a broader dynamic range than microarrays. Although, when using the same set of samples, both platforms have shown a high correlation between gene expression profiles (Zhao et al., 2014). In any case, RNA-seq data storage is particularly challenging and analysis is more complex when it comes to analyzing wheat genes belonging to what is likely the most complicated genome. The limited number of wheat studies based on RNA-seq is likely a consequence of its polyploidy, which complicates the step of sequence assembly and annotation, generating a bottleneck for data interpretation.

*Trichoderma* is a genus of filamentous fungi that includes strains that are able to colonize the rhizosphere and promote plant growth (Shoresh et al., 2010; Hermosa et al., 2012), and can help plants to overcome biotic and abiotic stress conditions (Rubio et al., 2014, 2017). The growth-promoting activity of *Trichoderma* isolates was already described in horticultural plants during the 80’s decade (Chang et al., 1986) and their beneficial effects have been attributed to different mechanisms including solubilization of several plant nutrients, root colonization and secretion of siderophores, phytohormones, vitamins and enzymes (Altomare et al., 1999; Viterbo et al., 2010; Li et al., 2015). However, the *Trichoderma*-plant cross-talk is a dynamic process that depends on the *Trichoderma* strain, the concentration used, the plant material, the developmental stage of the plant and the interaction time (Harman, 2006; Hermosa et al., 2012). Actually, the EU legislation allows the registration and commercialization of *Trichoderma* strains as biostimulants with the limitation of those strains already registered as biocontrol agents and included in the EU list of approved active substances (Annex I of Directive 98/8/EC).

Little is known about *Trichoderma*-wheat interactions, and transcriptional responses of wheat plants to strains belonging to this fungal genus. Here, we have studied the early global transcriptomic responses of wheat seedling roots to *Trichoderma harzianum* T34 under different N supplies, using wheat genome 61K microarrays. The former results indicate that under our experimental conditions, after 48 h interaction, strain T34 causes transcriptional changes to a larger extent than the N source, these being majorly related to an increase of defenses accompanied by reduced growth of wheat seedlings.

## Materials and Methods

### Microorganisms and Wheat Seeds

*Trichoderma harzianum* CECT 2413 (Spanish Type Culture Collection, Valencia, Spain), also referred to as strain T34, was grown on potato dextrose agar medium (PDA, Difco Laboratories, Detroit, MI, United States) and spores were harvested as previously described by Rubio et al. (2017).

Wheat seeds (*Triticum durum* “Dorondon”) were surface-sterilized by gentle sequential shaking in 2% (v/v) sodium hypochlorite for 20 min and 0.1N HCl for 2 min, and then rinsed four times with sterile distilled water. Stratification of the seeds was conducted for 2 days at 4°C.

### Plant Material and Total RNA Extraction

Wheat seeds were placed inside Phytatray II boxes (Sigma-Aldrich, Madrid, Spain) containing Murashige & Skoog medium (MS, Duchefa Biochemie BV, Haarlem, Netherlands), as previously described in Rubio et al. (2014), and boxes were kept at 25°C under gentle stirring for 72 h. Spores of T34 were used to inoculate flasks containing 100 mL of potato dextrose broth medium (PDB, Difco Laboratories) at a concentration of 10^6^ spores/mL. T34 cultures were then maintained at 25°C and 180 rpm for 16 h. Then, fungal germlings were harvested by centrifugation at 4000 × *g* for 10 min, washed, resuspended in sterile distilled water and used to inoculate, at a concentration of 10^5^ germlings/mL, Phytatray II boxes that contained 72-h-old wheat seedlings. Phytatray II boxes containing wheat seedlings without inoculation of fungus were grown in parallel as a control. After 24 h incubation at 25°C and gentle shaking, the lids were removed in sterile conditions and the MS medium was replaced with a minimal medium (Lullien et al., 1987) containing 0, 0.1, 0.5, or 1 mM Ca(NO_3_)_2_ as N source. The eight culture conditions assayed were coded as follows: T0N0 [without T34 strain and 0 mM Ca(NO_3_)_2_], T0N0.1 [without T34 strain and 0.1 mM Ca(NO_3_)_2_], T0N0.5 [without T34 strain and 0.5 mM Ca(NO_3_)_2_], T0N1 [without T34 strain and 1 mM Ca(NO_3_)_2_], T1N0 [T34 strain and without Ca(NO_3_)_2_], T1N0.1 [T34 strain and 0.1 mM Ca(NO_3_)_2_], T1N0.5 [T34 strain and 0.5 mM Ca(NO_3_)_2_] and T1N1 [T34 strain and 1 mM Ca(NO_3_)_2_]. These culture conditions were chosen after evaluating the growth of 72-h-old wheat seedlings for 48 h in the above described Phytatray boxes in minimal medium containing 0, 0.1, 0.5, 1, 2, 5, 7, or 10 mM Ca(NO_3_)_2_ (Supplementary Figure S1).

Roots from 10 wheat seedlings were pooled and homogeneized under liquid nitrogen. A fraction of 0.1 g of roots was taken and used for analyzing nitrate reductase activity, and the rest was kept at −80°C until total RNA extraction. At the same time, ten wheat seedlings from each 48 h-Phytatray culture were taken to measure length of aerial part, fresh weight and dry weight, after maintaining wheat seedlings at 65°C for 2 days. 48-h Phytatray cultures were performed in duplicate for each tested condition.

RNA from roots was extracted using TRIZOL reagent (Invitrogen, Gaithersburg, MD, United States), following manufacturer’s instructions. For microarray hybridizations, RNA was purified using the RNeasy MinElute Cleanup kit (Qiagen, Hilden, Germany). Purified total RNA samples were quantified with a NanoDrop^®^ ND-1000 (NanoDrop, Wilmington, DE, United States) spectrophotometer, and satisfactory purity was indicated by A260:280 ratios of 1.9–2.1. Integrity of total RNA samples was assessed by denaturing formaldehyde gel electrophoresis, where the presence of sharp 28S and 18S ribosomal RNA bands at an intensity ratio of ∼2:1 (28S:18S) indicated good integrity.

### Wheat Genome Array Hybridization and Data Analysis

cDNA was synthesized from 100 ng of purified RNA and used for production of labeled probes, which were used to hybridize the Affymetrix GeneChip Wheat Genome Array (Affymetrix, Santa Clara, CA, United States), employing the GeneChip Fluidics Station 450. The GeneChip Wheat Genome Array contains 61,127 probe sets representing 55,052 transcripts for all 42 chromosomes (21 in duplicate) in the wheat genome. 59,356 probe sets represent a modern hexaploid bread wheat (*Triticum aestivum*) and 1,759 probe sets derive from ESTs, 1,215 being from a diploid wheat (*T. monococcum*), 539 from a tetraploid macaroni wheat (*T. turgidum*), and five from a diploid known as *Aegilops tauschii*.

A total of 16 GeneChips were used in this study, two for each one of the eight assayed growth conditions. Purified RNA from roots of 10 wheat seedlings was labeled and used for hybridizing each GeneChip. All steps were done according to protocols described in the Gene Expression Analysis Technical Manual^1^. Briefly, following chips hybridization, the fluorescent signals emitted were scanned in a calibrated Affymetrix GeneChip Scanner 3000, and images analyzed in an Affymetrix GeneChip Operating Software (GCOS 1.2) for calculating the mean signal and for normalizing with the on-chip control genes using the Model Based Expression (dChip) Index (Li and Wong, 2001). The probe sets, showing differential expression [fold-change (FC) ≥ 2 and corrected *-* value < 0.015] in both biological replicates, were associated to wheat transcripts through a search in the IWGSC v0.4 genome and manually annotated using Gene Ontology (GO) terms (Ashburner et al., 2000), which were based on the BLAST definitions, applying an *E*-value < 10^–20^ level.

### Validation of Differential Expression by Quantitative Real-Time PCR

Seven genes, which showed significant differential expression from the microarray data, were selected for quantitative real-time PCR (qPCR) analysis. cDNA was synthesized from 1 μg total RNA using the Transcriptor First Strand cDNA Synthesis kit (Takara, Inc., Tokyo, Japan) with an oligo (dT) primer, following the manufacturer’s protocol. Then, 1 μL of cDNA was used in the subsequent PCR. All PCRs were performed in triplicate on a StepOne PlusTM device (Applied Biosystems, Foster City, CA, United States). Reaction mixtures and PCR amplification conditions were carried out as previously described (Rubio et al., 2017). Primers used are listed in Supplementary Table S1, and were designed using PerlPrimer software. The relative expression levels were calculated applying the 2^–ΔΔ^*^*C*^*^t^ method (Livak and Schmittgen, 2001) and using the actin transcript as an internal reference.

### Nitrate Reductase Assay

Protein extract was obtained from root material (0.1 g) that was grounded under liquid nitrogen. Extraction buffer (0.5 mL) containing 50 mM Tris (pH 8.5), 10 mM DTT, 0.02 mM Leupeptin, 0.01 mM FAD and 10 mM MgCl_2_ was added to the vegetal tissue, homogenized by vortexing, and supernatant containing proteins were recovered by centrifugation at 12,000 rpm for 10 min at 4°C. Nitrate reductase activity was determined following a previously described method (Abenavoli et al., 2016). Non-phosphorilated nitrate reductase activity was defined as μmol of nitrite produced in 1 min, and specific activity was such activity expressed per mg of protein. A standard curve was prepared using buffer, without NADH, with a final concentration of KNO_2_ ranging from 0 to 0.015 mM. The protein concentration was measured by Bradford assay using the Dye Reagent Concentrate (Bio-Rad Laboratories GmbH, München, Germany) and bovine serum albumin as a protein standard.

### Statistical Analyses

Data used for statistical analyses were obtained from at least three independent replicates, and expressed as the mean values. Statistical comparisons were evaluated by one-way ANOVA, followed by a *post hoc* Tukey’s test using the Statview 5.0 software. Confidence intervals of 95% were set.

## Results and Discussion

### Overview of the Wheat Gene Expression Data From Microarray Analysis

**FIGURE 1:**
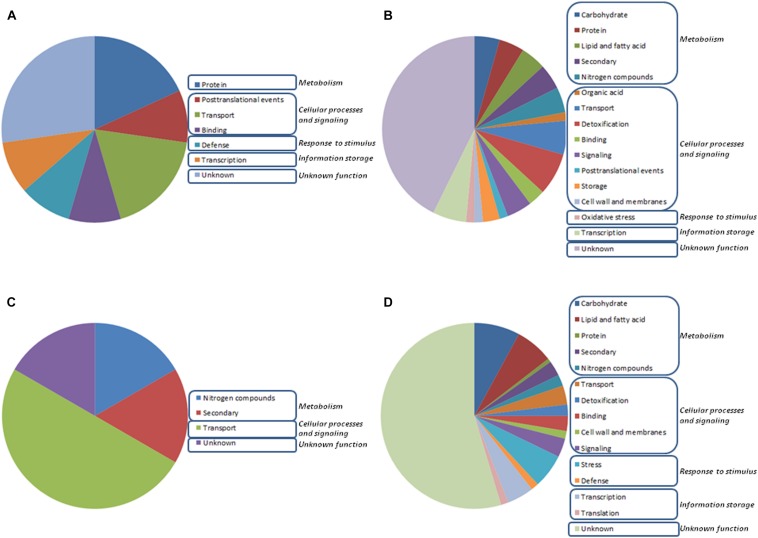
Distribution within physiological processes of the 226 differentially expressed genes at early stage (48 h growth) in wheat seedling roots resulting from the GeneChip Wheat Genome Array analysis is depicted for eight different growth conditions with (T1) or without (T0) *Trichoderma harzianum* T34 and 0 (N0), 0.1 (N0.1), 0.5 (N0.5) or 1 (N1) mM calcium nitrate. Gene significance was assigned to at least twofold change (*P*: 0.15). Comparisons were performed between **(A)** T0N0 and T0N0.5 or T0N1, **(B)** T0N0 and T1N0, **(C)** T1N0 and T1N0.5 or T1N1, and **(D)** T0N0.1, T0N0.5 or T0N1 and T1N0.1, T1N0.5 or T1N1.

A microarray-based transcriptome analysis was done using roots of wheat seedlings after 48 h culture under different N supplies, and colonized or not by *Trichoderma* with the aim of identifying genes that are differently regulated due to the presence of the fungus, the concentration of the N source or a combination of both parameters. In the present study, we have used wheat microarrays because this technology has become more available to us (Morán-Diez et al., 2012; Rubio et al., 2014), with well-established protocols and capacity to analyze data. Out of a total of 61,127 probe sets deposited on the microarray, only 226 genes showed at least a twofold significant change in expression using a *P*-value < 0.015 in at least one of the eight assayed conditions (Supplementary Table S2). No GO categories were significantly over-represented after comparing the expression changes detected in the eight considered growth conditions. For this reason, all the differentially expressed genes were analyzed independently and then manually grouped into physiological processes (Supplementary Table S2). The vast majority of the differentially expressed genes (50.9%) corresponded to hypothetical proteins with unknown function, without matches in databases, or to probes to which no transcript was found associated, despite having a hit in the wheat genome (Figure 1). A plausible explanation for this fact could be that the durum wheat is a polyploid plant, thus, obtaining a suitable sequence assembly and annotation is very complex due to occurrence of multiple copies of gene sequences (homologous or paralogous genes) (Singh et al., 2014). The largest number of the genes with a putative function were classified into “metabolism” (21.7%), most of them being involved in “carbohydrate metabolism” (6.6%), and a second gene set was grouped in “cellular processes and signaling” (19.5%), the majority of them corresponding to transport processes (6.2%) (Figure 1). The expression changes observed affecting such biological processes could be an indication of the active adaptation process of wheat seedlings to new growth conditions. Most of the genes exhibited expression changes only in one of the eight tested conditions and only 19 genes showed transcriptional changes in two or more conditions (Supplementary Table S2). Six out of the 19 genes encoded hypothetical proteins with unknown function or had no matches in databases. The other 13 were related to metabolism (five genes), cellular processes and signaling (six genes), response to stimulus (one gene) and information storage and processing (one gene).

**FIGURE 2:**
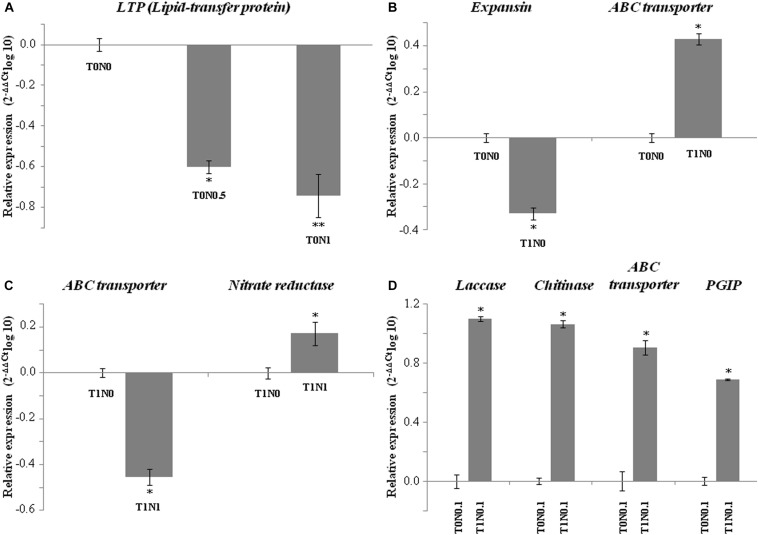
qPCR expression analysis of seven International Wheat Genome Sequencing Consortium (IWGSC, v0.4)-referred genes of a set of 226 genes differently expressed in wheat seedling roots resulting from the GeneChip Wheat Genome Array analysis under eight different growth conditions with (T1) or without (T0) *Trichoderma harzianum* T34 and 0 (N0), 0.1 (N0.1), 0.5 (N0.5) or 1 (N1) mM calcium nitrate. Analyzed gene code for: **(A)** Lipid-transfer protein (LTP, ID TraesCS1A01G175200.1) (T0N0.5 and T0N1 compared to T0N0 conditions), **(B)** expansin (ID TraesCS4A01G034300.1) and ABC transporter (ID TraesCS5A01G016200.1) (T1N0 compared to T0N0), **(C)** ABC transporter and nitrate reductase (ID TraesCS6B01G024900.1) (T1N1 compared to T1N0), and **(D)** laccase (ID TraesCS3B01G489800.1), chitinase (ID TraesCS7A01G371600.1), ABC transporter, and polygalacturonase inhibitor (PGIP, ID TraesCS7B01G103900.1) (T1N0.1 compared to T0N0.1). Data are the mean of three technical replicates from two biological replicates and are displayed as the log10 of the relative quantity (RQ, 2^– DDCt^) of target genes compared with the quantity of actin gene used as a reference. For each biological replicate, roots from ten plants were pooled.

To validate microarray results, qPCR assays were performed for seven genes within the set of 111 genes with putative function, that were differentially expressed in wheat roots under at least one of the eight growth conditions assayed (Figure 2). Non-specific lipid transfer protein gene represents those genes for which expression is dependent on Ca(NO_3_)_2_ concentration in the absence of *T. harzianum* T34, two genes coding for an expansin and an ABC transporter represent those differentially expressed in response to T34 strain in the absence of N source, and five genes are representative of those differentially expressed in growth conditions where T34 strain and Ca(NO_3_)_2_ combinations were applied. qPCR results correlated well with the data from the microarray experiment.

### Wheat Genes Differentially Expressed in Response to Ca(NO_3_)_2_ or *T. harzianum*

It has been recently described that Ca(NO_3_)_2_ concentrations higher than 5 mM suppressed cucumber seedlings growth (Fan et al., 2017). Thus, the N concentration effect on the growth of 72-h-old wheat seedlings was firstly evaluated, employing a Ca(NO_3_)_2_ concentration ranging from 0 to 10 mM. In terms of plant size after 48 h growth, the best result matched with 1 mM since 95% of plants had a size of 13 cm or larger, while a detrimental effect was observed when N concentration was increased (Supplementary Figure S1). It is noteworthy to mention that concentrations of 5, 7, or 10 mM Ca(NO_3_)_2_ gave rise to percentages lower than 35% plants belonging to the larger size group. Results obtained for wheat seedlings growth under the described experimental conditions are in a good agreement with those reported for cucumber (Fan et al., 2017). Therefore, in the current study, plant culture media containing a Ca(NO_3_)_2_ concentration ranging from 0 to 1 mM were used for exploring the effect of the N dosage on wheat seedling roots at a transcriptomic level. No differences in gene expression were detected between T0N0.1 and T0N0 conditions, thus indicating that, at least after 48 h Ca(NO_3_)_2_ application, a 0.1 mM concentration was not sufficient to cause transcriptomic changes in wheat seedling roots, in comparison with those that occurred in the absence of a N source. Four and ten genes, respectively, differed significantly in expression by at least twofold in T0N0.5 and T0N1 conditions when compared to the T0N0 condition (Supplementary Table S3). Most of them being down-regulated. Among them, three genes were common for both conditions (T0N0.5 and T0N1), encoding a putative ripening-related protein, a cysteine peptidase and a non-specific lipid transfer protein, respectively (Table 1). It has been reported that cysteine proteases are strongly expressed in wheat upon exposure to abiotic stresses, such as drought, heat or salinity conditions, which can also cause premature senescence (Botha et al., 2017). On the other hand, non-specific lipid transfer proteins are able to transfer lipids between membranes and it has been reported that they play several roles in plants, including defense against pathogens (Boutrot et al., 2005), as well as tolerance to salinity, cold and drought (Pitzschke et al., 2014). Similarly, a gene coding for an F-box protein was differentially down-regulated in the T0N1 condition, and it has been described that it is involved in wheat tolerance to abiotic stresses (Li et al., 2018). Thus, the down-regulation of these genes could be indicative of an absence of stress in wheat seedlings after 48 h growing under T0N0.5 or T0N1 conditions.

**TABLE 1:** Short list of annotated hits for wheat genes that alter their expression in response to *Trichoderma* and/or calcium nitrate selected due to their putative role in plant defense or development.

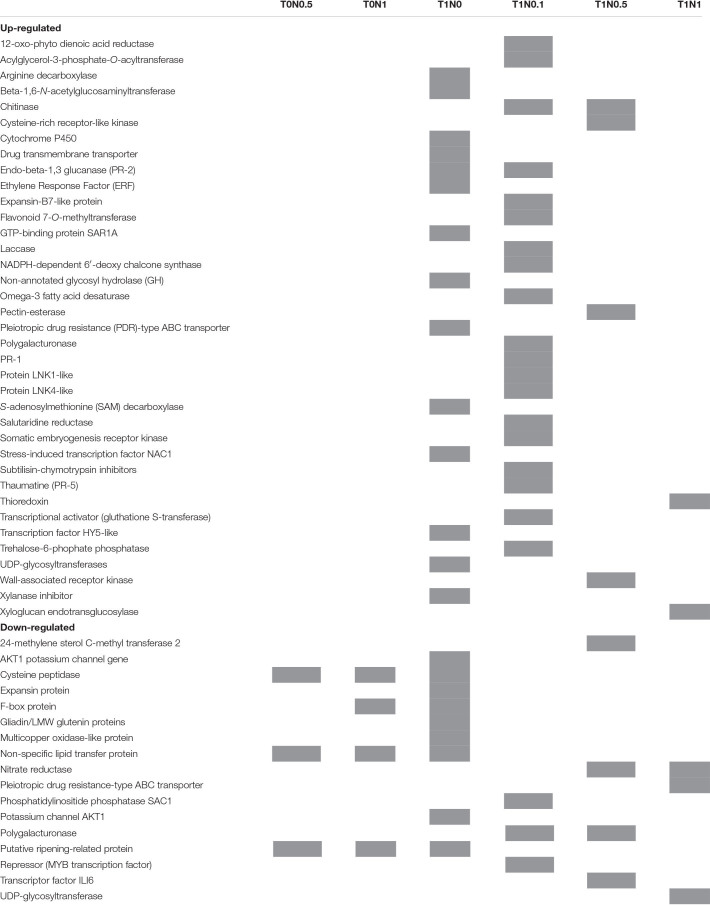

*Different growth conditions, including *Trichoderma harzianum* T34 (T1) or not (T0) and 0 (N0), 0.1 (N0.1), 0.5 (N0.5), or 1 (N1) mM calcium nitrate, were used for comparison.*

**FIGURE 3:**
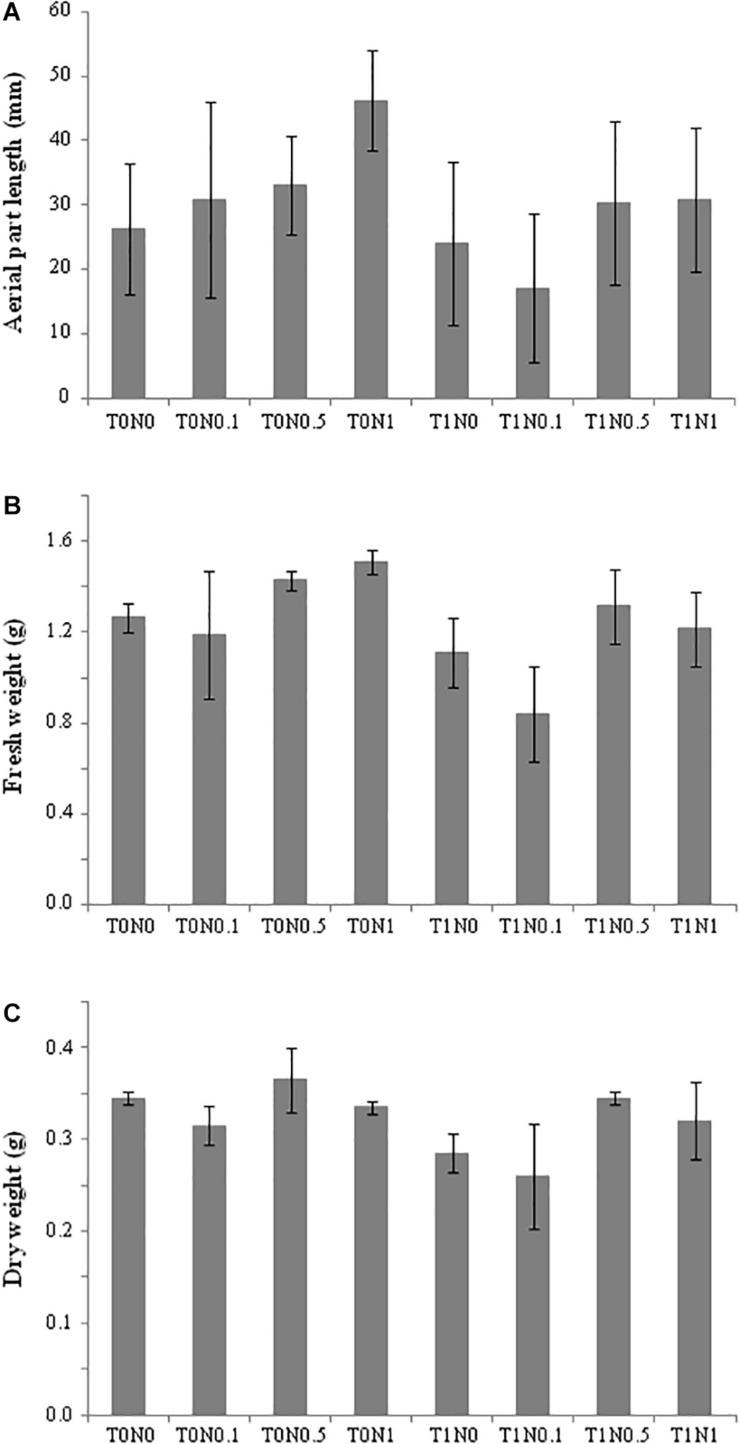
Measurements of wheat seedlings after being growth for 48 h under eight different conditions: with (T0) or without (T1) *Trichoderma harzianum* T34 and 0 (N0), 0.1 (N0.1), 0.5 (N0.5) or 1 (N1) mM calcium nitrate as nitrogen source. **(A)** Aerial part length, **(B)** fresh weight, and **(C)** dry weight. For each measurement, mean values of 20 plants from two independent experiments are shown.

Regarding *Trichoderma* application, a total of 67 genes differed significantly in their expression in root by at least twofold when T0N0 and T1N0 conditions were compared. They would be associated with the effect of the T34 strain in the absence of a N source (Supplementary Table S4): 55 genes were up-regulated, whereas 12 were down-regulated. “Metabolism” and “cellular processes and signaling” were the two most affected physiological processes. Interestingly, in spite of being grouped in different biological processes, several of the up-regulated genes were related to plant defense responses and tolerance to stress conditions. In this sense, numerous studies have shown that several transcription factors are important in regulating plant responses to environmental stress (Chen et al., 2002; Rubio et al., 2017). In the present study, four genes encoding transcription factors, two ethylene response factors (ERF), elongated hypocotyl 5 (HY5)-like and nucleus accumbens associated 1 (NAC1), showed higher expression levels in roots of the condition T1N0 than in T0N0 roots. *ERF* genes play a role in the ethylene (ET)-dependent defense pathway, which is activated upon infection with different types of pathogens, including bacteria, fungi and oomycetes (Gimenez-Ibanez and Solano, 2013). ET is also an important phytohormone for plant growth, development, senescence, and abiotic stress tolerance. In addition, it is described that ERFs are not only involved in response to ET signal transduction, but can also regulate ET biosynthesis in plant tissues (Hoang et al., 2017). HY5 is a master regulator of seedling development which modulates the expression of one third of the genes in *Arabidopsis* (Gangappa and Botto, 2016), being ERFs among them. HY5-ERF regulon restrains overproduction of ET, which is important for plant growth. According to this, it could be expected that T1N0 plants had a larger size than T0N0. In our study, length and fresh and dry weight values obtained from aerial wheat seedling parts, after 48 h growing in the presence of *T. harzianum* T34, were lower than those in absence of the fungus. This phetotype was reproducible in several independent experiments (Figure 3). An explanation might be the involvement of HY5 in the modulation of abscisic acid (ABA) and auxin pathways (Liu et al., 2018), and in the repression of cell elongation-responsive genes (Gangappa and Botto, 2016). In this sense, down-regulation of a gene coding an expansin 2 protein was detected in the condition T1N0 when compared to T0N0 (Figure 2). In wheat plants, expansins are involved in growth and developmental processes, as well as in tolerance to stress conditions, and their expression in other plants confers tolerance to salt, drought, cadmium and oxidative stresses (Zhang J.F. et al., 2018). In addition, down-regulation of a gene encoding a multicopper oxidase-like protein was also detected in the condition T1N0. It has been suggested that an homolog gene takes part in growth processes, possibly by participating in cell wall expansion in *Arabidopsis* (Sedbrook et al., 2002). In the same way, a reduced expression of *AKT1* potassium channel gene was detected. Potassium is a macronutrient for plants that is required for numerous physiological processes, such as membrane potential maintenance and turgor pressure, enzyme activation, osmotic pressure regulation, stomatal movement and tropism phenomena (Golldack et al., 2003). The induction of potassium channel genes by phytohormones like auxins gives them a central role in seedling growth and embryonic development. It has been shown that the potassium transporter AKT1 is required for the plant response to changes in external potassium, and subsequent regulation of potassium-dependent root growth and auxin redistribution in the root (Li J. et al., 2017).

**FIGURE 4:**
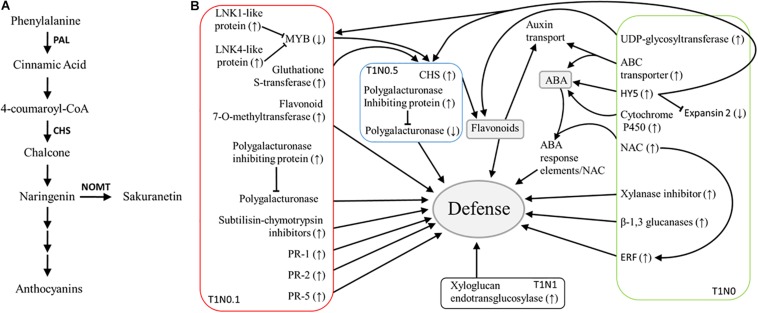
Schematic representation of defense activation by *Trichoderma harzianum* T34 in wheat seedlings. **(A)** Scheme of the major branch pathway of flavonoid biosynthesis, seven genes differently expressed in wheat seedling roots resulting from the GeneChip Wheat Genome Array analysis under different growth conditions with *Trichoderma harzianum* T34 and 0 (T1N0, green), 0.1 (T1N0.1, red), 0.5 (T1N0.5, blue) or 1 (T1N1, black) mM calcium nitrate belong to this pathway. Enzyme names are abbreviated as follows: Chalcone synthase (CHS), Naringenin 7-*O*-methyltransferase (NOMT) and Phenylalanine ammonia-lyase (PAL). **(B)** Signaling networks involving major genes related to plant-*Trichoderma* interaction that show upregulation (↑) or downregulation (↓) in the current study.

In another way, the transcription factor family NAC has been reported to have pivotal functions in mediating plant responses against various abiotic stresses due to their ability to specifically bind to the sequence of ABA-responsive elements (Hoang et al., 2017). Although more than 100 NAC members are present in the bread wheat genome, NAC1 is a novel member of this family, which has dual roles in response to pathogens, since it may modulate plant jasmonic acid (JA)- and salicylic acid (SA)-signaling defense cascades (Wang et al., 2015). The up-regulation of *NAC1* gene in wheat seedling roots from the condition, T1N0, is indicative of defense response activation mediated by T34 strain, at the same time compromising the plant growth. It can be thought that ABA-dependent defenses are triggered by T34 strain. In this sense, four genes encoding cytochrome P450 monooxygenase were up-regulated in the condition, T1N0. Proteins of this class have been involved in ABA biosynthesis (Narusaka et al., 2004). Also, a gene coding for a pleiotropic drug resistance (PDR)-type ABC transporter was up-regulated, which seems to play a role in ABA transport (Kang et al., 2011; Curci et al., 2017). Several ABC transporters were also up-regulated in durum wheat roots stressed by N starvation when analyzed through an RNA-sequencing approach (Curci et al., 2017). These changes detected at a transcriptomic level are in a good agreement with the phenotypic differences observed between wheat seedlings of the T1N0 and T0N0 conditions (Figure 3), because elements like HY5, cytochrome P450 monooxygenase and ABC transporter would be able to trigger NAC1-dependent defense pathways by means of ABA (Figure 4).

Three genes encoding decarboxylases, enzymes involved in N and amino acid metabolism, were up-regulated in wheat seedlings in response to T34. Two of them encoded arginine decarboxylases and the third one coded for a *S*-adenosylmethionine (SAM) decarboxylase. Both enzymes participate in polyamine biosynthesis, being the arginine decarboxylase involved in the formation of putrescine from arginine, and the SAM decarboxylase in the spermidine and spermine biosynthesis process from putrescine. The activation of polyamine metabolism correlates with the smaller growth of the T1N0 seedlings since spermine and spermidine induce concentration-dependent oxidative damage, resulting in decreased wheat biomass (Szalai et al., 2017). Recently, an up-regulation of tomato genes included in categories such as “spermine and spermidine biosynthetic process” and “arginine catabolic process” has been associated with *T. atroviride* P1-tomato interactions (Coppola et al., 2019). Accumulation of polyamines, as well as the up-regulation of genes coding for SAM decarboxylase in wheat plants, under environmental stress conditions have been previously described (Li and Chen, 2000), highlighting the role of polyamines in plant resistance to adverse circumstances. Polyamine profiles analyzed in *Trichoderma*-*Arabidopsis* interactions have been related to the *Trichoderma* species used, the timing of the interaction and whether it occurred with physical contact or not, which may confer upon these compounds a role reprogramming the changes undergone by plants to fine tune their defense and growth responses (Salazar-Badillo et al., 2015). The relationship between polyamines and phytohormones has been also established since ET induces SAM descarboxylase in plants (Van de Poel et al., 2013) and endogenous SA and polyamine content have been linked in wheat plants (Szalai et al., 2017).

Different defense mechanisms could be activated by strain T34 in the wheat seedling roots. Supporting this, an up-regulation of three genes encoding glycosyl hydrolase (GH) proteins involved in “carbohydrate metabolism” (an non-annotated GH, an endo-β-1,3 glucanase and a 1,6-*N*-acetylglucosaminyl transferase) was observed in the condition T1N0. GHs catalyze the hydrolysis of glycosidic bonds and have been described to participate in plant defense and development processes. Within GHs, β-1,3 glucanases belong to the GH family 17 and are considered to be pathogenesis-related (PR) proteins, since they are coordinately expressed in response to various biotic and abiotic stress stimuli (Thomas et al., 2000). The up-regulation of a gene encoding a xylanase inhibitor was also observed. This enzyme inhibits microbial endoxylanases belonging to the GH family 11 (Raedschelders et al., 2004), and it has a role in the protection of wheat plants against biotic or abiotic stresses (Pollet et al., 2009). The up-regulation of a gene coding for a calcium binding protein can be related to plant defense because its involvement in wheat defense response to pathogens and abiotic stresses has been described (Feng et al., 2011). Since an up-regulation of two genes encoding UDP-glycosyltransferases was observed in the condition T1N0, it can be assumed that T34 strain is triggering the anthocyanin biosynthesis pathway in wheat seedlings. Anthocyanins, a main class of flavonoids, play multiple roles in higher plants since they provide colors, absorb UV and high light irradiation, act as insect and animal attractants and are also antioxidants. Apart from that, they are effective agents against pathogens in plants (Li D. et al., 2017).

Wheat gluten comprises glutenin and gliadin proteins that account for up to 80% of the total protein content in the mature grain and determine viscoelastic properties of wheat flour dough, which govern the behavior of dough processing and baking quality (Wan et al., 2013). Genes encoding glutenin and gliadin proteins are regulated by N supply in developing wheat grain (Wan et al., 2013). Currently, wheat crop yield and quality rely on chemical nitrogenous fertilizers application (Zörb et al., 2018). The present microarray analysis showed no differential expression in such kinds of genes after comparing data from T0N0 and T0N0.1, T0N0.5 or T0N1 conditions (Supplementary Table S3). However, a down-regulation of two genes encoding gliadin/Low Molecular Weight (LMW) glutenin proteins was detected in the condition T1N0 when compared with T0N0. Since we are analyzing changes in a root transcriptome, it is not possible to forecast that T34 strain will cause a decrease in gluten protein content of wheat grain. Recently, proteins with a gliadin domain have been identified as being involved in wheat plant immunity against *F. graminearum* (Zhang Y. et al., 2018). The observed down-regulation of glutenin genes in response to T34 may indicate that this strain of *T. harzianum* would not be triggering this novel plant defense mechanism.

The observed transcriptomic changes, together with the T1N0 seedling phenotype (Figure 3) would seem contradictory to some attributes associated with the beneficial effects of *Trichoderma*. Although, we ought not forget that the transcriptome had been analyzed when the *Trichoderma* colonization was still active. It would be reasonable to speculate that wheat seedlings, at least locally, and in response to T34 strain, increase defense to limit the fungal penetration to the outer layers of root cortical cells, being compromised the plant growth in return. Moreover, the up-regulation of *ERF*, *NAC1* and *HY5* genes in plants inoculated with *Trichoderma* indicates that, at least through these regulation hubs, the fungus adjusted the phytohormone networking leading to activate defenses, which in party explains the reduced size of the wheat seedlings treated with T34.

### Wheat Genes Differentially Expressed in Response to Ca(NO_3_)_2_ and *Trichoderma* Combinations

The root transcriptional response of wheat seedlings to the combined application of *T. harzianum* T34 and different concentrations of N source (T1N0.1, T1N0.5 or T1N1 conditions) was analyzed, either taking as reference T1N0 (Supplementary Table S5) or T0N0.1, T0N0.5 and T0N1 conditions (Supplementary Table S6). The huge difference in the number of the differentially expressed genes between both comparatives, seven and 157, respectively, would be a good indicator of the important role that *Trichoderma* plays in the transcriptomic changes detected in wheat roots.

#### N Source Effect When Applied in Combination With *T. harzianum* T34

Only seven genes showed differential expression after comparing T1N0.1, T1N0.5 or T1N1 conditions with the condition T1N0. No differences in gene expression were detected when the conditions, T1N0 and T1N0.1, were compared. There were two and five genes that differed significantly in expression by at least twofold in wheat seedling roots between T1N0 and T1N0.5 or T1N1, respectively (Supplementary Table S5). One of the up-regulated genes (ID TraesCS6B01G024900.1), which was shared by both conditions (T1N0.5 and T1N1), codes for nitrate reductase. This enzyme catalyzes the reduction of nitrate to nitrite, which is highly toxic to plant cells and is reduced to ammonium by the enzyme nitrite reductase and finally, this ammonium can be used by the plant to produce amino acids and proteins (Krapp, 2015). Surprisingly, genes coding for N metabolism enzymes were not detected in response to an increase of the N source, that is when comparing T0N0.1, T0N0.5 or T0N1 conditions with the condition T0N0. However, this scenario changes when T34 strain is present, thus indicating that the fungus is able to modulate the plant N metabolism, at least at the two highest N concentrations tested. In order to confirm these microarray data, nitrate reductase activity was measured in the same root material employed for microarray analysis, as well as in the aerial part of wheat seedlings (Table 2). The highest nitrate reductase values were obtained in roots from T1N0.5 condition, likewise the aerial part of seedlings from T1N0.5 condition also gave the highest levels of this activity. Similar results were obtained in independent experiments. It has already been shown that changes in N concentration govern plant N metabolism, allowing them to mitigate the acclimatory effect to adverse environments (Vicente et al., 2016). Another up-regulated gene in the condition T1N1, with respect to T1N0, codes for a thioredoxin. Wheat thioredoxins have been involved in germination and seedling development by promoting mobilization of primary storage proteins, inactivation of small amylolytic enzyme inhibitors, and activation of calcium-dependent substrate-specific proteases (Pilcher et al., 2017). Previous studies have reported the induction of genes coding for thioredoxins by the herbicide metribuzin in wheat (Pilcher et al., 2017).

**TABLE 2:** Specific nitrate reductase activity, expressed as μmol⋅min^–1^ per mg protein, measured in either aerial part or root from wheat seedlings after growing for 48 h under eight different conditions: with or without *Trichoderma harzianum* T34 and 0, 0.1, 0.5, or 1 mM calcium nitrate as nitrogen source.

**Conditions**	**Aerial part**	**Root**
T0N0	13.43 ± 1.54^a^	11.78 ± 1.06^ab^
T0N0.1	12.10 ± 1.35^ab^	11.18 ± 1.12^ab^
T0N0.5	13.51 ± 1.06^a^	10.58 ± 0.61^ab^
T0N1	14.02 ± 0.99^a^	10.43 ± 0.87^ab^
T1N0	9.40 ± 2.47^b^	9.50 ± 1.01^b^
T1N0.1	11.83 ± 1.78^ab^	10.01 ± 1.39^ab^
T1N0.5	12.27 ± 0.71^a^	12.20 ± 2.19^a^
T1N1	12.88 ± 1.05^a^	9.88 ± 1.03^b^

*Values are means of three technical replicates from two biological replicates with the corresponding standard deviation. For each biological replicate, roots or aerial part from ten plants were pooled. Values in the same column with different letters are significantly different according to Tukey’s test (*P* < 0.05).*

Three more genes, encoding an UDP-glycosyltransferase and two PDR-type ABC transporters, were down-regulated in seedling roots from T1N1 condition in comparison with T1N0 condition, one out of two transporter genes being up-regulated in roots of the condition T1N0 compared with T0N0 condition. These data were confirmed by qPCR analysis (Figure 2). The differences detected for this ABC transporter, for some of the assayed conditions, suggest that the scenario can be drastically changed when different N source concentrations and *Trichoderma* are applied in combination. Thus, great care must be taken for selecting the right combination to obtain the desired outcome.

#### *T. harzianum* T34 Effect When Applied in Combination With Different N Inputs

The larger number of transcriptomic changes observed among all different conditions tested for wheat seedling roots was in the presence of T34 strain combined with the lowest N supply. Almost 80% of differently expressed genes were up-regulated. Just in the presence of T34 strain without N source (T1N0), 67 genes were differentially expressed, but when N was included this number was higher, reaching 73 genes in the comparison between T1N0.1 and T0N0.1 conditions (Supplementary Table S6). Although less numerous, a great variety of changes, 61 and 23 genes, were detected when comparing T1N0.5 and T0N0.5 or T1N1 and T0N1 conditions, respectively. More than the half of the genes (56%) that differentially varied in their expression when T34 strain was applied in combination with different N supplies corresponded to hypothetical proteins with unknown function or had no matches in databases. The largest number of genes with putative function were associated with physiological processes included within the “metabolism” category (20.4%), being mainly affected the carbohydrate metabolism but also differential expression changes were detected for several genes related to lipid and fatty acid metabolism. The second largest set of genes was located within the “cellular processes and signaling” category (15.3%). In addition to the expected genes from subcategories, such as “transport” or “response to stimulus,” it is noteworthy to mention that six genes from the “signaling” subcategory were up-regulated. Most of these last six genes code for kinase receptor proteins (Supplementary Table S6). On the basis of these results, we can conclude that the *Trichoderma* effect is more striking when low concentrations of Ca(NO_3_)_2_ are supplied, existing a correlation between an increase of N source applied in combination with T34 strain and a reduction in the number of transcriptomic changes in root of wheat seedlings, at least after 48 h of growing.

Overall, many of the genes differentially expressed in wheat seedling roots, due to a combined application of T34 and Ca(NO_3_)_2_ as an N source, have already been described as components of plant defense responses against pathogens. In wheat, PR-protein activation has been described in a resistant genotype as a defense response to infection by *F. graminearum*, in comparison with susceptible lines to this pathogen (Golkari et al., 2009). An up-regulation of endo-β-1,3 glucanase (PR-2) and chitinase genes was detected in wheat seedlings from T1N0.1 and T1N0.5 conditions. In addition, genes encoding proteins, such as PR-1, thaumatine (PR-5), and polygalacturonase and subtilisin-chymotrypsin inhibitors, which have been previously described and are involved in wheat defense against pests and pathogens (Caruso et al., 1999; Golkari et al., 2009), were differentially up-regulated in the T1N0.1 condition. In parallel to wheat defense activation, detected in response to the application of strain T34 combined with the lowest N concentration, the up-regulation of genes related to plant development was also detected. This is the case for the gene encoding trehalose-6-phophate phosphatase, an enzyme involved in trehalose production, which was up-regulated in T1N0.1 condition. It is well known that trehalose has a protector role in plant against abiotic stresses by preventing the denaturation of cellular proteins (Fernandez et al., 2010). However, trehalose-6-phosphate has emerged as an important signaling metabolite, regulating carbon assimilation and the sugar status in plants, thus having an essential role in development (Ponnu et al., 2011). Several genes that are also involved in cell wall structure modulation (Zhang J.F. et al., 2018) were up-regulated when T34 was applied along with different N concentrations. This is the case for genes coding for pectin-esterase (ID TraesCS3B01G258100.1) (T1N0.5), expansin-B7-like protein (ID TraesCS4B01G375600.1) (T1N0.1), laccase (ID TraesCS3B01G489800.1) (T1N0.1), and xyloglucan endotransglucosylase (ID TraesCS2A01G433500.1) (T1N1). On the other hand, some genes coding for proteins like polygalacturonase (ID TraesCS3B01G020300.1) (T1N0.1 and T1N0.5), 24-methylene sterol C-methyl transferase 2 (ID TraesCS1A01G204700.1) (T1N0.5), phosphatidylinositol phosphatase SAC1 (ID TraesCS1A01G020700.1) (T1N0.1), and the transcription factor ILI6 (ID TraesCS4A01G016300.1) (T1N0.5), which are involved in plant growth and development (Yang et al., 2018), were down-regulated. Transcriptomic analysis shows that the *Trichoderma* effect is more evident when it is applied in combination with low N supply. In this sense, the lowest number of differentially expressed genes was detected in the condition, T1N1, in comparison with those detected in T1N0.1 or T1N0.5. The hability of T34 strain to modulate plant defense and growth responses is remarkable.

It is noteworthy to mention that, in the condition T1N0.5, an important number of up-regulated genes were associated with the major physiological processes “signaling” or “response to stimulus stress” (Table 1 and Supplementary Table S6), being annotated as receptor kinase or ABA-responsive protein, respectively. Such up-regulation observed in wheat roots is in good agreement with a previous study, in which up-regulation of ABA-related genes was described in *Arabidopsis* after 24 h of incubation in the presence of *T. harzianum* T34 (Morán-Diez et al., 2012). It is known that ABA plays a key role in the activation of plant cellular adaptation to abiotic stresses, and that, under adverse environmental conditions, this phytohormone functions as a growth inhibitor. Moreover, it is accepted that mitogen-activated protein kinase (MAPKs) mediates signal transduction cascades, they being central integrators of plant abiotic stress signaling since they link to cellular signaling, and metabolic and stress adaptative processes in the plant (Golldack et al., 2014). In view that an evident up-regulation of ABA-responsive element and receptor kinase genes was detected in the T1N0.5 condition, together with the above described up-regulation of ABA-related genes in T1N0, it could be thought that MAPKs and ABA also have a central role in plant defense responses to *Trichoderma* (Figure 4), linking primary metabolism and developmental processes. According to this scenario, it has recently been suggested that future plant holistic studies must consider biotic stress, abiotic stress and development processes (Bigeard and Hirt, 2018).

In a previous study, down-regulation of induced systemic resistance-linked genes was observed in *Arabidopsis* roots colonized by the endopolygalacturonase *Thpg1*-silenced transformant of *T. harzianum* T34 (Morán-Diez et al., 2009). Chalcone synthase (CHS) and xyloglucan endotransglucosylase genes were among the 10 *Arabidopsis* genes that were significantly affected by the silencing of *Thpg1* in T34, and in the present work, these two genes (Table 1) were up-regulated in wheat seedling roots from the conditions, T1N0.5 and T1N1, respectively. CHS is a member of the plant polyketide synthase superfamily, and it is the key enzyme of the flavonoid biosynthesis pathway, providing the starting materials for the synthesis of these compounds (Dao et al., 2011) (Figure 4). An up-regulation of the CHS gene, accompanied by a down-regulation of a polygalacturonase gene, was observed in wheat seedling roots from the treatment T1N0.5. Moreover, genes involved in the flavonoid biosynthesis, such as flavonoid 7-*O*-methyltransferase gene, were differentially expressed in T1N0.1 condition (Table 1). In this regard, the up-regulation of genes encoding transcriptional activators (i.e., glutathione S-transferase) and coactivators (i.e., LNK1- and LNK4-like proteins) was observed, as well as the down-regulation of repressor genes (i.e., MYB transcription factor), indicative of an increased resistance in wheat seedling roots. In addition to the accumulation of flavonoids in plants, which have an important role in auxin regulation by inhibiting its polar transport, CHS is involved in the SA defense pathway. It is known that plants activate SA biosynthesis in response to *Trichoderma* root colonization to prevent the fungus from entering the vascular system (Alonso-Ramírez et al., 2014). Regarding flavonoids, it is worth highlighting that several genes involved in their biosynthesis were differentially expressed under T1N0, T1N0.1, and T1N0.5 conditions, thus reinforcing the relevance of such pathway during plant–*Trichoderma* interaction.

## Conclusion

The early root transcriptomic changes observed in wheat seedling roots colonized by the *T. harzianum* T34 under different N supplies show that *Trichoderma* contributes to a larger extent than the N source to such changes. When the fungus is applied in combination with low N concentrations, the plant transcriptional changes are led to balance growth with the need for defense. Based on the fact that many changes observed affect the flavonoid biosynthesis pathway, we assume that *Trichoderma* induces plant defenses through such mechanism.

## Data Availability Statement

The microarray data are available at the GEO database with accession number GSE134903.

## Author Contributions

RH and EM conceived the research. MR, CN, and RH performed the experiments. MR and RH analyzed the data. EM and RH contributed reagents, materials, and analysis tools. MR, AM, and RH wrote the manuscript with the contributions from all other authors on the discussion of the data.

## Conflict of Interest

The authors declare that the research was conducted in the absence of any commercial or financial relationships that could be construed as a potential conflict of interest.

## Footnotes

1 http://www.affymetrix.com

## Supplementary Material

FIGURE S1 Calcium nitrate [Ca(NO_3_)_2_] effect on the size of 72-h-old wheat seedlings after 48 h growing in a Phytatray box system. Aboveground plant length was measured for each Ca(NO_3_)_2_ concentration tested (0, 0.1, 0.5, 1, 2, 5, 7, and 10 mM), and as a result, three groups were set up: larger or equal than 13 cm, from 10 to 13 cm, and shorter or equal than 10 cm. Plant height percentages for each growth condition within either of the three groups are depicted by bars. The number of plants used to calculate these percentages is indicated above each bar. **(A)** Ca(NO_3_)_2_: 0, 2, 5, 7 and 10 mM, *n* = 30, and **(B)** Ca(NO_3_)_2_: 0, 0.1, 0.5, 1 and 2 mM, *n* = 20.

TABLE S1 Primers used for quantitative real-time PCR (qPCR) analysis.

TABLE S2 List of genes that showed at least a twofold significant change in expression using a *P-*value < 0.015 in at least one of the eight assayed conditions (as described in Materials and Methods).

TABLE S3 Physiological processes differentially affected in wheat seedling roots in response to the nitrogen concentration in the plant growth media, 0.5 or 1 mM calcium nitrate [Ca(NO_3_)_2_] compared to nitrogen source absence.

TABLE S4 Physiological processes differentially affected in wheat seedling roots in response to *Trichoderma harzianum* T34 in the absence of nitrogen source.

TABLE S5 Physiological processes differentially affected in wheat seedling roots in response to the combined application of *Trichoderma harzianum* T34 and 0.5 or 1 mM calcium nitrate [Ca(NO_3_)_2_] in the plant growth medium compared to nitrogen source absence.

TABLE S6 Physiological processes differentially affected in wheat seedling roots in response to the combined application of *Trichoderma harzianum* T34 and 0.1, 0.5, or 1 mM calcium nitrate [Ca(NO_3_)_2_] in the plant growth medium compared to T34 strain absence.
