# Supplementary figures and images for: Early Root Transcriptomic Changes in Wheat Seedlings Colonized by Trichoderma harzianum Under Different Inorganic Nitrogen Supplies
Source: Front Microbiol. 2019 Oct 25;10:2444. doi: 10.3389/fmicb.2019.02444 (PMC6842963; doi:10.3389/fmicb.2019.02444)

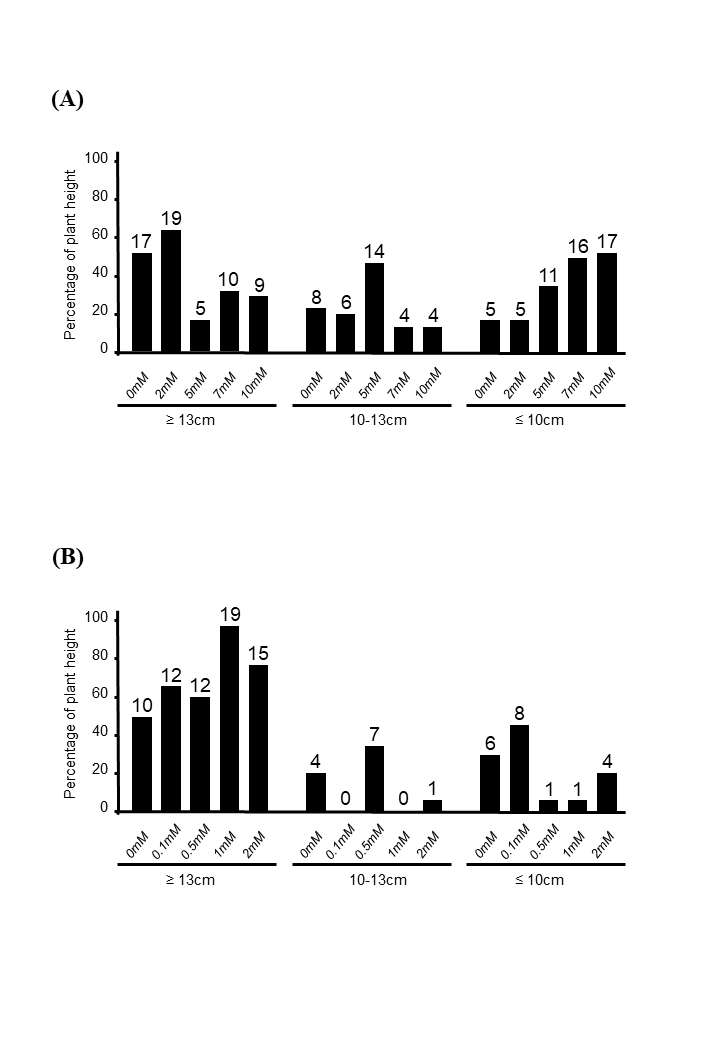

Supplement: FIGURE S1 — Calcium nitrate [Ca(NO3)2] effect on the size of 72-h-old wheat seedlings after 48 h growing in a Phytatray box system. Aboveground plant length was measured for each Ca(NO3)2 concentration tested (0, 0.1, 0.5, 1, 2, 5, 7, and 10 mM), and as a result, three groups were set up: larger or equal than 13 cm, from 10 to 13 cm, and shorter or equal than 10 cm. Plant height percentages for each growth condition within either of the three groups are depicted by bars. The number of plants used to calculate these percentages is indicated above each bar. (A) Ca(NO3)2: 0, 2, 5, 7 and 10 mM, n = 30, and (B) Ca(NO3)2: 0, 0.1, 0.5, 1 and 2 mM, n = 20. [file Image_1.TIF]
