# Supplementary material for: Early Root Transcriptomic Changes in Wheat Seedlings Colonized by Trichoderma harzianum Under Different Inorganic Nitrogen Supplies
Source: Front Microbiol. 2019 Oct 25;10:2444. doi: 10.3389/fmicb.2019.02444 (PMC6842963; doi:10.3389/fmicb.2019.02444)
Supplement: TABLE S1 — Primers used for quantitative real-time PCR (qPCR) analysis. [file Table_1.DOCX]

**Table S1.** Primers used for quantitative real-time PCR (qPCR) analysis.

| **Name** | **Sequences (5’ → 3’)** | **Hit description** | **References** |
| --- | --- | --- | --- |
| LTP-Fw | CGTGCTGCAACAACCTAAAGAG | Non-specific lipid-transfer protein | This work |
| LTP-Rv | GGGCTGTTGATGTAGCTGGAGTA |  |  |
| Expansin-Fw | AGATGGGAGGGATCAGGTTCA | Expansin | This work |
| Expansin-Rv | GATCAGCACCAGCTCGAAGTT |  |  |
| ABC-Fw | GAGCAGTATCGTCACGTGTCTGT | PDR-type ABC transporter | This work |
| ABC-Rv | TCTGCTGGCCTACGTGGAA |  |  |
| nia-Fw | CTCAAGCGCAGCACGTCTA | NADH-nitrate reductase | Vicente *et al.* (2016) |
| nia-Rv | CTCGGACATGGTGAACTGCT |  |  |
| LAC-Fw | GGAGCTCGACCTCGTTGAGT | Laccase | This work |
| LAC-Rv | CGATAGAGGATTGTCGTCGAAGT |  |  |
| CHIT-Fw | CCAGGCTCTGTGGAGGAAGTA | Chitinase | This work |
| CHIT-Rv | TGACGTTGCCGGTCTTGA |  |  |
| PGI-Fw | CCATCCCGCCACTCTTCTAC | Polygalacturonase inhibiting protein | This work |
| PGI-Rv | AGGTGGTTGTTGGAGAGCAAA |  |  |
| Actin-Fw | TGACCGTATGAGCAAGGAG | Actin | This work |
| Actin-Rv | CCAGACACTGTACTTCCTC |  |  |
