# Supplementary material for: Early Root Transcriptomic Changes in Wheat Seedlings Colonized by Trichoderma harzianum Under Different Inorganic Nitrogen Supplies
Source: Front Microbiol. 2019 Oct 25;10:2444. doi: 10.3389/fmicb.2019.02444 (PMC6842963; doi:10.3389/fmicb.2019.02444)
Supplement: TABLE S3 — Physiological processes differentially affected in wheat seedling roots in response to the nitrogen concentration in the plant growth media, 0.5 or 1 mM calcium nitrate [Ca(NO3)2] compared to nitrogen source absence. [file Table_3.docx]

**Table S3.** Physiological processes differentially affected in wheat seedling roots in response to the nitrogen concentration in the plant growth media, 0.5 or 1 mM calcium nitrate [Ca(NO_3)2_] compared to nitrogen source absence.

| **Up-regulated** | | | |
| --- | --- | --- | --- |
| **Physiological process** | **Hit description** | **Probe sets 0.5 mM [Ca(NO_3)2_] (*Fold change*)** | **Probe sets 1 mM [Ca(NO_3)2_] (*Fold change*)** |
| Metabolism |  |  |  |
| Protein | Aspartic-type endopeptidase |  | Ta.16530.1.A1_at (+2.73) |
|  |  |  |  |
| Cellular processes and signaling |  |  |  |
| Posttranslational events | Oxidoreductin protein |  | Ta.1995.3.S1_a_at (+2.04) |
|  |  |  |  |
| Information storage and processing |  |  |  |
| Transcription | LNK4-like protein (transcriptional coactivator) |  | Ta.25294.1.S1_at (+6.12) |
|  |  |  |  |
| Unknown function |  | Ta.25004.1.A1_at (+2.41) |  |
| **Down-regulated** | | | |
| **Physiological process** | **Hit description** | **Probe sets 0.5 mM CN (*Fold change*)** | **Probe sets 1 mM CN (*Fold change*)** |
| Metabolism |  |  |  |
| Protein | Cysteine peptidase | Ta.13737.1.S1_s_at (-5.88) | Ta.13737.1.S1_s_at (-5.30) |
|  |  |  |  |
| Cellular processes and signaling |  |  |  |
| Transport | Non-specific lipid-transfer protein | TaAffx.97181.1.S1_s_at (-3.05) | TaAffx.97181.1.S1_s_at (-5.39)  Ta.1526.1.S1_x_at (-2.65) |
| Binding | F-box protein |  | Ta.21439.1.S1_s_at (-2.03) |
|  |  |  |  |
| Response to stimulus |  |  |  |
| Defense | Putative ripening related protein | Ta.1215.1.S1_at (-9.95) | Ta.1215.1.S1_at (-8.62) |
|  |  |  |  |
| Unknown function |  |  | Ta.14033.2.S1_at (-2.13)  Ta.16251.2.S1_x_at (-2.05) |
