# Supplementary material for: Early Root Transcriptomic Changes in Wheat Seedlings Colonized by Trichoderma harzianum Under Different Inorganic Nitrogen Supplies
Source: Front Microbiol. 2019 Oct 25;10:2444. doi: 10.3389/fmicb.2019.02444 (PMC6842963; doi:10.3389/fmicb.2019.02444)
Supplement: TABLE S4 — Physiological processes differentially affected in wheat seedling roots in response to Trichoderma harzianum T34 in the absence of nitrogen source. [file Table_4.DOCX]

**Table S4.** Physiological processes differentially affected in wheat seedling roots in response to *Trichoderma harzianum* T34 in the absence of nitrogen source.

| **Up-regulated** | | |
| --- | --- | --- |
| **Physiological process** | **Hit description** | **Probe sets (*Fold change*)** |
| Metabolism |  |  |
| Carbohydrate | Glycosyl hydrolase | Ta.6243.1.A1_at (+2.29) |
|  | Endo-β-1,3 glucanase | Ta.21297.1.S1_at (+2.70) |
|  | 1,6-N-acetylglucosaminyl transferase | Ta.8970.1.A1_s_at (+2.43) |
| Protein | Subtilisin-like protease | Ta.26151.1.A1_at (+4.80)  Ta.8949.1.A1_at (+3.86) |
|  | Xylanase inhibitor | Ta.20434.2.S1_x_at (+2.60) |
| Lipid and fatty acid | Enoyl-CoA hydratase | Ta.9392.2.A1_at (+2.19) |
|  | Pyruvate decarboxylase | Ta.13281.1.S1_at (+2.21) |
| Secondary | UDP-glycosyltransferase | TaAffx.42793.1.A1_x_at (+7.20)  Ta.15081.1.S1_at (+2.38) |
|  | Phenolic glucosidase malonyltransferase | Ta.12059.1.S1_at (+3.14) |
| Amino acid and Nitrogen compounds | S-adenosylmethionine decarboxylase | Ta.9063.3.S1_at (+3.33) |
|  | Arginine decarboxylase | Ta.22221.1.S1_x_at (+2.02)  Ta.22221.1.S1_a_at (+2.3) |
| Organic acid | 2-oxoglutarate-Fe (II) type oxidoreductase | Ta.3564.2.S1_a_at (+5.74) |
|  |  |  |
| Cellular processes and signaling |  |  |
| Transport | Drug transmembrane transport | Ta.5289.1.S1_at (+2.06) |
|  | GTP-binding protein SAR1A | TaAffx.37720.1.S1_at (+3.27) |
|  | Pleiotropic drug resistance (PDR)-type ABC transporter | TaAffx.70601.1.S1_at (+7.73) |
| Detoxification | Cytochrome p450 monooxygenase | Ta.21061.1.S1_x_at (+3.42)  Ta.19609.1.S1_at (+2.00)  Ta.19295.1.S1_at (+2.00)  TaAffx.50125.2.S1_at (+6.13) |
| Binding | Calcium-binding protein | TaAffx.29126.1.S1_at (+6.04) |
| Signaling | Carbonic anhydrase | Ta.10295.1.S1_at (+2.86)  Ta.10295.1.S1_x_at (+2.65) |
| Posttranslational events | Oxidoreductin protein | Ta.1995.3.S1_a_at (+2.23) |
|  |  |  |
| Response to stimulus |  |  |
| Oxidative stress | Cationic peroxidase SPC4 | Ta.21307.2.S1_x_at (+3.39) |
|  |  |  |
| Information storage and processing |  |  |
| Transcription | Ethylen-responsive transcription factor (ERF) | Ta.22338.2.S1_a_at (+5.87)  Ta.21056.1.S1_at (+5.75) |
|  | Stress-induced transcription factor SNAC1 | Ta.5367.1.S1_s_at (+2.53) |
|  | Transcription factor HY5-like | TaAffx.38635.1.A1_at (+3.80) |
| Unknown function |  | * |
| **Down-regulated** | | |
| **Physiological process** | **Hit description** | **Probe sets (*Fold change*)** |
| Cellular processes and signaling |  |  |
| Transport | Potassium channel AKT1 | Ta.241.1.S1_at (-2.01) |
| Storage | Gliadin/LMW glutenin | Ta.905.2.S1_x_at (-3.81)  TaAffx.42864.3.S1_x_at (-3.46) |
| Detoxification | Cytochrome p450 monooxygenase | Ta.15927.1.S1_at (-2.07) |
| Binding | Multicopper oxidase-like protein | Ta.3391.1.A1_s_at (-2.16) |
| Cell wall and membranes | Expansin | TaAffx.86321.1.S1_at (-2.38) |
| Signaling | Two-component response regulator ARR8 | TaAffx.119928.1.A1_s_at (-3.10) |
|  |  |  |
| Unknown function |  | ** |

^*^ Ta.5861.2.S1_at (+58.61), Ta.21061.2.S1_at (+19.01), Ta.97.1.S1_at (+9.32), Ta.21061.2.S1_x_at (+8.53), Ta.27299.1.S1_x_at (+6.64), Ta.8405.1.A1_at (+4.74), Ta.24665.2.S1_at (+3.72), Ta.19858.1.A1_x_at (+3.37), Ta.19041.1.S1_at (+3.28), Ta.23066.1.S1_s_at (+3.06), TaAffx.15836.1.S1_at (+3.06), Ta.5673.1.S1_at (+2.65), TaAffx.5215.1.S1_at (+2.62), TaAffx.132318.2.S1_at (+2.55), Ta.22556.1.S1_x_at (+2.55), Ta.15199.1.S1_at (+2.30), TaAffx.115983.1.S1_at (+2.2), Ta.5825.1.S1_x_at (+2.12), TaAffx.6514.1.S1_x_at (+2.07), Ta.23309.1.S1_at (+2.06), TaAffx.78755.1.S1_at (+2.04), TaAffx.7266.1.S1_at (+2.03), TaAffx.15261.3.S1_at (+2.03), TaAffx.6514.1.S1_at (+2.02).

^**^ TaAffx.129103.1.S1_at (-55.45), Ta.4006.1.A1_at (-2.72), TaAffx.50907.3.S1_s_at (-2.19), TaAffx.12816.1.A1_at (-2.08), Ta.7239.1.A1_at (-2.08).
