# Supplementary material for: Early Root Transcriptomic Changes in Wheat Seedlings Colonized by Trichoderma harzianum Under Different Inorganic Nitrogen Supplies
Source: Front Microbiol. 2019 Oct 25;10:2444. doi: 10.3389/fmicb.2019.02444 (PMC6842963; doi:10.3389/fmicb.2019.02444)
Supplement: TABLE S5 — Physiological processes differentially affected in wheat seedling roots in response to the combined application of Trichoderma harzianum T34 and 0.5 or 1 mM calcium nitrate [Ca(NO3)2] in the plant growth medium compared to nitrogen source absence. [file Table_5.docx]

**Table S5.** Physiological processes differentially affected in wheat seedling roots in response to the combined application of *Trichoderma harzianum* T34 and 0.5 or 1 mM calcium nitrate [Ca(NO_3)2_] in the plant growth medium compared to nitrogen source absence.

| **Up-regulated** | | | |
| --- | --- | --- | --- |
| **Physiological process** | **Hit description** | **Probe sets T34+0.5 mM [Ca(NO_3)2_] (*Fold change*)** | **Probe sets T34+1 mM [Ca(NO_3)2_] (*Fold change*)** |
| Metabolism |  |  |  |
| Nitrogen compounds | Nitrate reductase | Ta.5633.1.S1_a_at (+2.17) | Ta.5633.1.S1_a_at (+2.02) |
|  |  |  |  |
| Cellular processes and signaling |  |  |  |
| Transport | Thioredoxin |  | Ta.19627.1.S1_at (+2.05) |
| **Down-regulated** | | | |
| **Physiological process** | **Hit description** | **Probe sets T34+0.5 mM CN (*Fold change*)** | **Probe sets T34+1 mM CN (*Fold change*)** |
| Metabolism |  |  |  |
| Secondary | UDP-glycosyltransferase |  | Ta.15081.1.S1_at (-2.64) |
|  |  |  |  |
| Cellular processes and signaling |  |  |  |
| Transport | Pleiotropic drug resistance (PDR)-type ABC transporter |  | Ta.8232.1.A1_at (-2.23)  TaAffx.70601.1.S1_at (-2.22) |
|  |  |  |  |
| Unknown function |  | TaAffx.31187.1.S1_at (-2.75) |  |
