# Supplementary material for: Early Root Transcriptomic Changes in Wheat Seedlings Colonized by Trichoderma harzianum Under Different Inorganic Nitrogen Supplies
Source: Front Microbiol. 2019 Oct 25;10:2444. doi: 10.3389/fmicb.2019.02444 (PMC6842963; doi:10.3389/fmicb.2019.02444)
Supplement: TABLE S6 — Physiological processes differentially affected in wheat seedling roots in response to the combined application of Trichoderma harzianum T34 and 0.1, 0.5, or 1 mM calcium nitrate [Ca(NO3)2] in the plant growth medium compared to T34 strain absence. [file Table_6.docx]

**Table S6.** Physiological processes differentially affected in wheat seedling roots in response to the combined application of *Trichoderma harzianum* T34 and 0.1, 0.5 or 1 mM calcium nitrate [Ca(NO_3)2_] in the plant growth medium compared to T34 strain absence.

| **Up-regulated** | | | |  | |
| --- | --- | --- | --- | --- | --- |
| **Physiological process** | **Hit description** | **Probe sets T34+0.1 mM [Ca(NO_3)2_] (*Fold change*)** | **Probe sets T34+0.5 mM [Ca(NO_3)2_] (*Fold change*)** | **Probe sets T34+1 mM [Ca(NO_3)2_] (*Fold change*)** |  |
| Metabolism |  |  |  |  |  |
| Carbohydrate | Beta-1,3 glucanase | Ta.19591.1.A1_at (+2.62)  TaAffx.9022.1.S1_at (+5.95) | TaAffx.119315.2.S1_at (+4.34)  TaAffx.119315.2.S1_x_at (+5.15)  Ta.21051.1.S1_at (+5.64) |  |  |
|  | Chitinase | Ta.6051.3.S1_x_at (+6.34)  Ta.224.1.S1_at (+4.36) |  |  |  |
|  | Trehalose-phosphate phosphatase | TaAffx.58849.1.S1_at (+2.59) |  |  |  |
|  | Polygalacturonase inhibiting protein | TaAffx.37424.1.A1_at (+3.03) | TaAffx.37424.1.A1_at (+3.65) |  |  |
|  | Xyloglucan endotransglucosylase |  |  | Ta.13337.1.S1_at (+2.42) |  |
| Lipid and fatty acid | Esterase/lipase | Ta.8585.1.S1_at (+2.32) |  |  |  |
|  | 2-C-methyl-D-erythritol 2.4-cyclodiphosphate synthase | Ta.8196.1.S1_a_at (+2.77) |  |  |  |
|  | Omega-3 fatty acid desaturase | Ta.24254.1.S1_a_at (+2.29) |  |  |  |
|  | Acylglycerol-3-phosphate O-acyltransferase |  | Ta.26236.1.A1_at (+37.70) |  |  |
|  | Flavonoid7-O methyltransferase | Ta.14165.1.S1_at (+36.53) |  |  |  |
|  | 12-oxo-phyto dienoic acid reductase |  | Ta.8780.1.A1_at (+2.37) |  |  |
| Protein | Subtilisin-chymotrypsin inhibitor | Ta.14230.1.S1_at (+4.59) |  |  |  |
| Secondary | Laccase | Ta.25771.1.A1_at (+12.67) |  |  |  |
|  | UDP-glycosyltransferase | Ta.1120.1.S1_x_at (+2.32) |  |  |  |
|  | NADPH-dependent 6’-deoxy chalcone synthase |  | Ta.9399.1.S1_at (+4.19) |  |  |
| Nitrogen compounds | Pheophorbide A oxigenase | Ta.2728.1.S1_at (+2.02) |  |  |  |
|  | Salutaridine reductase |  | Ta.10615.1.A1_at (+3.95) |  |  |
|  |  |  |  |  |  |
| Cellular processes and signaling |  |  |  |  |  |
| Transport | Pleiotropic drug resistance (PDR)-type ABC transporter | TaAffx.70601.1.S1_at (+8.47)  Ta.9385.1.A1_at (+4.00)  Ta.8232.1.A1_at (+8.77) | Ta.9831.1.S1_s_at (+2.50) |  |  |
|  | UDP-glucose transporter | Ta.4921.1.S1_at (+2.46) |  |  |  |
| Detoxification | Cytochrome p450 monooxygenase | Ta.8346.1.A1_at (+3.86) |  |  |  |
|  | Glutathione S-transferase |  | Ta.303.2.S1_x_at (+3.23)  Ta.14632.1.S1_at (+2.47) |  |  |
| Binding | Calcium-binding protein KIC-like | Ta.15067.1.S1_x_at (+2.0) |  |  |  |
|  | Heme-binding-like protein | Ta.5969.3.S1_x_at (+2.33)  Ta.5969.2.S1_x_at (+2.29)  Ta.5969.1.A1_at (+2.06) |  |  |  |
| Cell wall and membranes | Pectinesterase |  | TaAffx.5634.1.S1_at (+2.57) |  |  |
|  | Expansin-B7-like protein | Ta.24423.1.S1_s_at (+2.23) |  |  |  |
| Signaling | Two-component response regulator PRR73 | Ta.9719.1.S1_at (+3.55) | Ta.9719.1.S1_at (+3.03) |  |  |
|  | Serine/Threonine PK |  | TaAffx.77715.1.S1_at (+2.09) |  |  |
|  | Cysteine-rich receptor-like kinase |  | Ta.25487.1.S1_at (+4.17) |  |  |
|  | Somatic embryogenesis receptor kinase | Ta.27314.1.S1_at (+8.30) |  |  |  |
|  | Wall-associated receptor kinase |  | Ta.8759.1.S1_at (+2.11) |  |  |
|  |  |  |  |  |  |
| Response to stimulus |  |  |  |  |  |
| Stress | Senescence protein | Ta.12666.1.S1_at (+4.83) |  |  |  |
|  | Protein SRG-1 (stress response genes) |  | TaAffx.100029.1.S1_at (+3.16)  Ta.22674.1.A1_at (+2.24) |  |  |
|  | Abscisic stress-ripening protein ASR |  | Ta.1907.1.A1_at (+12.99) |  |  |
|  | ABA-responsive protein |  | Ta.27945.1.S1_x_at (+2.42) |  |  |
|  | Early salt stress and cold acclimation induced protein |  | Ta.351.1.S1_at (+2.56) |  |  |
|  | Ice recrystallization inhibition protein |  | Ta.21768.1.S1_x_at (+3.94)  Ta.21768.1.S1_at (+3.87) |  |  |
| Defense | PR pathogenesis-related protein-1 (PR-1) |  |  | Ta.62.1.S1_x_at (+5.79) |  |
|  | Thaumatin-like protein | Ta.25053.1.S1_at (+2.86) |  |  |  |
|  |  |  |  |  |  |
| Information storage and processing |  |  |  |  |  |
| Transcription | LNK4-like protein (transcriptional coactivator) | Ta.25294.1.S1_at (+5.24) |  |  |  |
|  | LNK1-like protein (transcriptional coactivator) | Ta.4689.1.S1_at (+2.79) |  |  |  |
| Translation | 50s ribosomal protein L31 |  |  | Ta.2429.3.S1_x_at (+17.29) |  |
|  |  |  |  |  |  |
| Unknown function |  | * | ** | *** |  |
| **Down-regulated** | | | |  | |
| **Physiological process** | **Hit description** | **Probe sets T34+0.1 mM CN (*Fold change*)** | **Probe sets T34+0.5 mM CN (*Fold change*)** | **Probe sets T34+1 mM CN (*Fold change*)** | |
| Metabolism |  |  |  |  | |
| Carbohydrate | Beta-1,3 glucanase | Ta.10197.1.S1_at (-4.27) |  |  | |
|  | Polygalacturonase | TaAffx.124202.2.S1_at (-3.01) | TaAffx.124202.2.S1_at (-3.20) |  | |
| Lipid and fatty acid | Esterase/lipase | TaAffx.7956.1.S1_at (-2.64) | Ta.29866.1.S1_at (-2.40) |  | |
|  | 24-methylen sterol C-methyl transferase 2 |  | TaAffx.128583.1.S1_at (-2.46) |  | |
|  | Phosphatidylinositide phosphatase SAC1 | TaAffx.38139.2.A1_at (-2.92) |  |  | |
| Secondary | UDP-glucosyl transferase | Ta.16173.1.S1_at (-4.35) |  |  | |
| Nitrogen compounds | Nicotianamine synthase |  |  | Ta.5145.3.S1_x_at (-4.55) | |
|  |  |  |  |  | |
| Information storage and processing |  |  |  |  | |
| Transcription | Transcription factor BHLM150-like | Ta.18938.1.S1_at (-4.90) |  |  | |
|  | Transcription factor ILI6 |  | TaAffx.29848.1.S1_at (-2.41) |  | |
|  | MYB transcription factor | Ta.25744.1.S1_at (-2.04) |  |  | |
| Translation | RNA-binding protein ARP1 | TaAffx.98159.1.S1_s_at (-3.28) |  |  | |
|  |  |  |  |  | |
| Unknown function |  | **** | ***** | ****** | |

^*^ Ta.9401.1.S1_a_at (+13.56), Ta.9401.2.S1_x_at (13.13), Ta.9401.3.S1_x_at (+12.10), Ta.9401.3.S1_at (+10.61), Ta.97.2.S1_x_at (+7.84), TaAffx.7297.1.S1_at (+7.26), Ta.8582.2.S1_x_at (+6.31), Ta.24738.1.S1_x_at (+5.13), Ta.22339.1.S1_at (+5.05), Ta.5824.2.S1_x_at (+3.63), TaAffx.124483.1.A1_at (+3.51), TaAffx.3542.1.S1_at (+3.51), Ta.13922.1.S1_at (+3.39), TaAffx.12271.2.S1_at (+3.25), Ta.9216.2.A1_at (+3.24), TaAffx.56225.1.S1_at (+2.95), Ta.8196.1.S1_x_at (+2.77), Ta.9216.2.A1_x_at (+2.75), Ta.24254.2.S1_at (+2.44), Ta.8196.3.S1_at (+2.43), Ta.8196.3.S1_x_at (+2.40), Ta.18507.1.S1_s_at (+2.09).

^**^ TaAffx.106983.1.S1_at (+544.78), Ta.17550.1.S1_s_at (+309.27), TaAffx.108468.1.S1_at (+142.09), Ta.30885.1.S1_at (+126.09), Ta.30865.1.S1_at (+106.80), TaAffx.24129.1.S1_at (+73.31), TaAffx.85840.1.S1_at (+70.49), Ta.25657.1.S1_at (+64.31), TaAffx.85702.1.S1_at (+42.06), Ta.17721.1.S1_s_at (+33.67), TaAffx.86581.2.S1_x_at (+27.29), TaAffx.86581.1.S1_s_at (+23.04), TaAffx.112284.1.S1_at (+17.43), TaAffx.112285.1.S1_at (+17.36), Ta.14184.1.S1_at (+15.57), Ta.4717.1.A1_at (+14.68), TaAffx.107944.1.S1_s_at (+13.24), Ta.5861.2.S1_at (+11.27), Ta.10603.1.S1_at (+7.48), TaAffx.37179.1.A1_at (+6.57), TaAffx.6469.1.S1_at (+6.30), Ta.1912.1.S1_at (+4.28), TaAffx.109595.1.S1_at (+3.28), Ta.8496.1.A1_at (3.28), Ta.13965.1.S1_at (+2.90), TaAffx.56225.1.S1_at (+2.88), Ta.7948.1.S1_at (+2.07), Ta.30251.1.A1_at (+2.06).

^***^ Ta.30865.1.S1_at (+382.59), Ta.9678.1.S1_at (+366.92), Ta.5946.1.S1_at (+154.56), TaAffx.30254.1.S1_at (+153.13), TaAffx.108468.1.S1_at (+80.40), TaAffx.57634.2.S1_at (+49.40), TaAffx.25796.1.S1_at (+27.24), TaAffx.78858.1.S1_at (+21.57), Ta.30822.1.S1_at (+21.25), TaAffx.112805.1.S1_at (+19.16), Ta.14184.1.S1_at (+18.26), Ta.15072.1.A1_at (+11.59), TaAffx.37179.1.A1_at (+9.76), Ta.10599.1.A1_at (+8.76), Ta.21556.1.S1_x_at (+5.09), TaAffx.12101.1.S1_at (+4.77), Ta.23399.1.S1_x_at (+2.84), Ta.23339.1.S1_at (2.15).

^****^ Ta1579.1.A1_at (-3.85), TaAffx.57166.1.S1_at (-3.61), TaAffx.12592.2.S1_at (-3.24), Ta.10328.1.S1_at (-2.85), Ta.21003.2.S1_a_at (-2.68), TaAffx.86713.1.S1_at (-2.64), Ta.7595.1.A1_at (-2.40), Ta.24624.1.S1_at (+2.36), TaAffx.113545.1.S1_at (-2.33), Ta.12877.1.S1_at (-2.33), Ta.30479.1.A1_at (-2.19), Ta.29552.1.S1_a_at (-2.16), Ta.12944.1.S1_at (-2.16).

^*****^ Ta20727.2.S1_x_at (-6.68), TaAffx.78327.1.S1_at (-2.76), TaAffx.109232.2.S1_at (-2.63), TaAffx.112166.1.S1_at (-2.61), TaAffx.128541.42.S1_at (-2.48), TaAffx.128541.17.S1_x_at (-2.37).

^******^ TaAffx.23355.1.S1_at (-2.32).
